# Stable Isotope Tracing Metabolomics to Investigate the Metabolic Activity of Bioactive Compounds for Cancer Prevention and Treatment

**DOI:** 10.3390/cancers12082147

**Published:** 2020-08-03

**Authors:** Feroza K. Choudhury, G. Lavender Hackman, Alessia Lodi, Stefano Tiziani

**Affiliations:** 1 Department of Nutritional Sciences, College of Natural Sciences, The University of Texas at Austin, Austin, TX 78712, USA; feroza.choudhury@austin.utexas.edu (F.K.C.); lavenderhackman@utexas.edu (G.L.H.); alessia@austin.utexas.edu (A.L.); 2 Department of Pediatrics, Dell Medical School, The University of Texas at Austin, Austin, TX 78723, USA; 3 Department of Oncology, Dell Medical School, The University of Texas at Austin, Austin, TX 78723, USA

**Keywords:** bioactive compounds, natural products, metabolic pathways, stable isotope tracing, cancer prevention, cancer metabolism

## Abstract

A major hallmark of cancer is the metabolic reprogramming of cancer cells to fuel tumor growth and proliferation. Various plant-derived bioactive compounds efficiently target the metabolic vulnerabilities of cancer cells and exhibit potential as emerging therapeutic agents. Due to their safety and common use as dietary components, they are also ideal for cancer prevention. However, to render their use as efficient as possible, the mechanism of action of these phytochemicals needs to be well characterized. Stable isotope tracing is an essential technology to study the molecular mechanisms by which nutraceuticals modulate and target cancer metabolism. The use of positionally labeled tracers as exogenous nutrients and the monitoring of their downstream metabolites labeling patterns enable the analysis of the specific metabolic pathway activity, via the relative production and consumption of the labeled metabolites. Although stable isotope tracing metabolomics is a powerful tool to investigate the molecular activity of bioactive compounds as well as to design synergistic nutraceutical combinations, this methodology is still underutilized. This review aims to investigate the research efforts and potentials surrounding the use of stable isotope tracing metabolomics to examine the metabolic alterations mediated by bioactive compounds in cancer.

## 1. Introduction

In co-ordination with deregulated cell proliferation in cancer, the energy metabolism is adjusted to the increased demand to fuel cell growth and division as well as to maintain the redox balance. Reprogramming energy metabolism is one of the major hallmarks of cancer [1,2,3]. Reprogrammed metabolism in cancer cells presents as important target for developing therapeutic regimens and preventative measures against cancer. Due to their diverse structure and the ability to target different aspects of the metabolism, plant-derived natural bioactive compounds have gained increasing interest over the years for combating cancer [4]. They have inspired the successful development of new drugs in the pharmaceutical industry [5]. Their diverse biological activity, bioavailability and tolerability have proven them to be potential therapeutic agents and a safe option for preventing cancer [6,7]. For their successful utilization against cancer, their mechanism of action needs to be well characterized. In this review, we discuss the natural bioactive compounds that target different aspects of cancer metabolism and techniques to investigate their mechanism of action.

## 2. Bioactive Compounds

**Figure 1:**
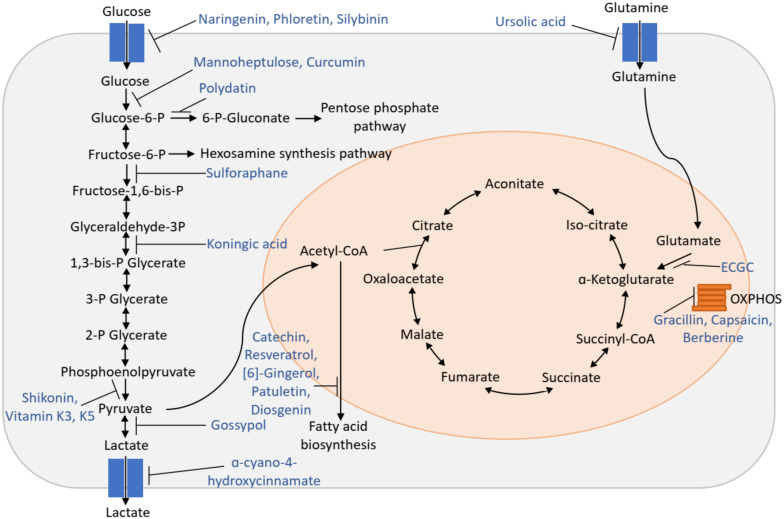
Different bioactive compounds that target metabolic processes. The primary target step of the bioactive compounds is shown and numbered in red color. 1. Glucose transporter (GLUT), 2. hexokinase, 3. glucose-6-phosphate dehydrogenase, 4. phosphofructokinase, 5. gyceraldehyde-3-phosphate dehydrogenase, 6. pyruvate kinase M2, 7. lactate dehydrogenase, 8. monocarboxylate transporter, 9. glutamine transporter, 10. glutamate dehydrogenase, 11. fatty acid synthase.

Different components of the altered metabolism in cancer can be targeted by bioactive compounds (Table 1, Figure 1). They can be used for treatment in various cancers and some have proven their efficacy in clinical trials. Preliminary studies have been done in cell culture experiments to analyze cytotoxic potential and mechanisms of action, followed by experiments in animal models to study tumor growth inhibitory potential. A few studies have been done in patients to determine therapeutic efficacy; however, additional patient data are necessary to fully elucidate the metabolic activity of these compounds in humans and more clinical studies should be performed in the future. The main principle by which these compounds work against cancer is by producing energy deficiency or by limiting the components needed for cell growth and proliferation. The combination of two compounds can have synergism and can be more effective in treatment against cancer. Ursolic acid in combination with curcumin and resveratrol has a synergistic effect and shows more potency in reducing tumor size in mouse allograft model of prostate cancer than when administered individually [8].

## 3. Inhibitors of Glucose Uptake

Naringenin inhibits insulin-stimulated glucose uptake in breast cancer cells by binding to estrogen receptor beta. It disrupts the insulin-induced GLUT4 translocation from intracellular compartment to the plasma membrane [9]. Naringenin has therapeutic potential as it reduced insulin-mediated glucose uptake and suppressed proliferation at a dose of 10µM [10]. Naringenin has anti-proliferative, proapoptotic anti-cancerous role in several cancer cell lines, such as breast (MDA-MB-231) [11], prostate (PC3, LNCaP) [12], melanoma (B16F10) [13], liver (HepG2) [14], HER2 positive breast cancer cell line [15] and mammary tumor cells (E0771) [16]. In rats bearing Walker 256 carcinosarcoma (W256), a dose of 25 mg/kg of naringenin inhibited tumor growth by around 75% [17]. The therapeutic potential of naringenin is greatly limited due to its low aqueous solubility and inefficient transport across biological membrane causing lower bioavailability in tumor sites [18]. D- α-tocopheryl polyethelene glycol succinate 1000 (TPGS)-coated naringenin nanosuspension can reverse the drug resistance and have higher cytotoxic potential in MCF-7 cells [19].

Flavonoids, such as myricetin, fisetin, quercetin, and its glucoside analog isoquercitin, inhibit GLUT2 present in the intestine. The non-competitive inhibition was observed at IC_50_ for quercetin, myricetin and isoquercitin that is approximately 200- to 1000- fold less than glucose and fructose concentration [20].

Phloretin, a dihydrochalcone, retarded tumor growth in vitro and in vivo by inhibiting glucose transporter GLUT1 and GLUT2 [21,22,23]. Phloretin can sensitize cancer cells to chemotherapeutic agents, such as daunorubicin, and mediates apoptosis to overcome drug resistance in colon cancer and leukemia cells in hypoxia [24]. In HepG2 xenografted mice, tumor growth was inhibited following combined treatment with phloretin and paclitaxel [25]. Phloretin inhibited proliferation and induced apoptosis in Calu-1, H838 and H520 cells and suppress the migration and invasion of NSCLC cells and enhanced the effect of cisplatin [26]. Phloretin increased the efficacy of HSP70 penetration and thus its anticancer activity in B16 mouse melanoma cells and K-562 human erythroblasts [27]. Phloretin showed anticancer activity in several human cancer cell lines, including lung (A549), liver (HepG2), colon (HT-29) [28], gastric (AGS) [29], esophageal (EC-109) [30], breast (MDA-MB-231) [31], prostate (LNCaP) [32] and glioblastoma cells [33]. Phloretin has a cancer preventative role by its activity in reducing oxidative DNA damage, as observed in human colon cancer cell lines (Caco-2 and HT-29) [34]. The preventative action is mainly mediated by elevated glutathione level by inducing rate limiting enzyme of glutathione biosynthesis, gamma-glutamyl cysteine ligase [35].

Sylibinin and its oxidized form 2,3-dihydrosilybinin inhibit cellular glucose uptake in different cancer cells by means of interaction with GLUT4, rendering an inhibitory effect on their proliferation and survival. 2,3-dihydrosilybinin has a stronger inhibition on glucose uptake. Sylibinin and 2,3-dihydrosilybinin inhibit GLUT4-mediated glucose transport in a competitive manner with K_i_ = 60 and 116 µM, respectively [36]. Sylibinin was found to be effective in clinical trials in prostate cancer patients [37,38]. Sylimarin, a family of flavonoids including sylibinin, can suppress the proliferation of several cancer cell lines, such as prostate, breast, ovary, colon, lung and bladder cancer cell lines, by arresting the cell cycle at G1/S- phase, through the induction of cyclin-dependent kinase inhibitors (such as p15, p21 and p27), down-regulation of anti-apoptotic gene products (e.g., Bcl-2 and Bcl-xL) and inhibition of cell-survival kinases (AKT, PKC and MAPK) [39,40,41]. Silymarin’s chemo preventive role has been associated with the inhibition of ultraviolet B radiation or chemically initiated carcinogenesis in skin cancer. Moreover, silymarin suppressed 3, 2-dimethyl-4-aminobiphenyl-induced carcinogenesis in prostate cancer and the growth of advanced prostate tumor xenografts in athymic nude mice [42].

Curcumin causes decreased protein levels of GLUT1 in breast and lung cancer cells and hexokinase II in colorectal cancer cells, which causes a decrease in glucose uptake, lactate production, and ATP generation [43,44]. Curcumin also caused dissociation of hexokinase II from mitochondria, causing mitochondria-mediated apoptosis [44]. Curcumin helped to overcome the resistance of triple negative breast cancer to 4-hydroxytamoxifen by inhibiting hexokinase II expression and promoting mitochondrion-mediated apoptosis [45]. It also induces apoptosis in HepG2 and MDA-MB-231 cells via inhibiting fatty acid synthase [46,47]. Apart from these, curcumin has shown anticancer potential in several other cancers, like pancreatic, hepatic, gastric, colorectal, prostate cancer [48,49].

## 4. Inhibitor of Glycolysis

Mannoheptulose is a seven-carbon sugar that inhibits hexokinase, thus reducing glycolysis, and could be used as a potential anti-cancer treatment [38,50].

Sulforaphane has anti-cancer activity in several cancer cell lines. The anti-cancer activity is mediated by down-regulation of 6-phosphofructo-2-kinase/fructose-2,6-biphosphatase4 (PFKFB4) protein, which is a key modulator of glycolysis. Sulforaphane also down regulates the expression of transcription factor, hypoxia inducible factor- 1α (HIF-1α), which regulates the expression of PFKFB4 [51]. The anti-cancer activity of sulforaphane is not mediated by direct inhibition of phosphofructokinase, but through the inhibition of the PFKFB4 pathway. It inhibits the growth of several cancer cell lines, including breast, prostate, colon, skin, lung, gastric and bladder cancer cell lines, by inducing cell cycle arrest and apoptosis [52]. It also has a cytoprotective role by activating Nrf2 mediated signaling pathway required for response to oxidative stress [53]. A phase II study with sulforaphane in men with recurrent prostate cancer showed moderate improvement in outcomes, and given the safety of the compound, higher doses were proposed to further improve treatment outcome [54]. In patients with advanced pancreatic ductal carcinoma, 90 mg/day of sulforaphane inhibited tumor growth and increased the sensitivity of cancer cells to chemotherapeutics [55].

Koningic acid is an inhibitor of glyceraldehyde 3-phosphate dehydrogenase (GAPDH) and the inhibition of its activity in NG108-15 cells leads to apoptosis [56]. It is a potent cytotoxic agent in both hypoxic and normoxic condition [57]. Inhibition of GAPDH and thus glycolysis by koningic acid can be effective against glycolytic cancer cells, which are more sensitive to glycolytic inhibition than their healthy counterparts [58].

Shikonin and its enantiomer alkannin are both potent inhibitors of pyruvate kinase M2 (PKM2) isoform with IC_50_ values of 0.8 and 0.9 µM, respectively. They are the most potent and specific inhibitors of PKM2 reported. In cell lines such as MCF7 and A549 tumor cells, which predominantly express PKM2, shikonin and alkannin caused decrease in both glucose consumption and lactate production, with significantly lower PKM2 activity in the cell lysate, which in total represent lower glycolytic flux [59]. Shikonin dose-dependently inhibited glucose uptake and lactate production in pre-B cell acute lymphoblastic leukemia (BCP-ALL) [60], Lewis lung carcinoma (LLC) and B16 melanoma cells and suppressed cellular aerobic glycolysis through inhibiting PKM2 by reducing PKM2 phosphorylation. Shikonin promoted tumor cell (B16) apoptosis in vitro and in vivo [61]. PKM2 is strongly upregulated in bladder cancer, which contributes to cisplatin resistance. Administration of shikonin to cisplatin resistant T24 cells inhibited PKM2 and re-sensitized them to cisplatin treatment [62]. Higher expression of PKM2 is also observed in esophageal cancer cells, which go through apoptosis and have reduced tumor burden when treated with shikonin [63].

Vitamin K3 and K5 mediates their anticancer effect by inhibiting PKM2, among them vitamin K5 is more potent. They both caused lower glycolytic flux and decreased survival of Hela cells [64].

Oleanolic acid is a potential anticancer agent as it induces a switch from PKM2, which is responsible for enhanced aerobic glycolysis in cancer cells, to PKM1 and thus abrogate the Warburg effect in cancer cells [65].

Gossypol is a non-selective inhibitor of lactate dehydrogenase (LDH) which competes with NADH. It has K_i_ of 1.9 and 1.4 µM for LDH-A and LDH-B, respectively. It also inhibits other NAD^+^ dependent enzymes, such as GAPDH [66]. The (R)-(-)-gossypol is a more potent cytotoxic agent with a mean IC_50_ value of 20 µM compared to (S)-(-)-gossypol. It exerts its cytotoxic effect on several cancer cell lines, including melanoma, lung, breast, cervix and leukemia [67]. Gossypol suppressed the growth of temozolomide-resistant glioblastoma cells [68]. It induced apoptosis in multiple myeloma [69], colon cancer (HT-29) [70], chronic lymphocytic leukemia [71] and prostate cancer (LAPC4, PC3, and DU145) with an IC_50_ between 35– µM and inhibited prostate tumor growth in a xenograft model [72]. Gossypol also inhibited the adhesion and invasion of human breast cancer cell lines [73].

α-cyano-4-hydroxycinnamate (αCHC) and other cinnamic acid derivatives are most studied monocarboxylate transporter (MCT) inhibitors [74,75]. αCHC inhibits MCT1 with a K_i_ of 166 µM, MCT2 with a K_i_ of 24 µM and MCT4 with a K_i_ of 991 µM [76]. Human melanoma cells, when treated with αCHC, had reduced intracellular pH at low extracellular pH, which resulted in reduced survival of these cells [77]. Treatment of malignant glioma cells with αCHC resulted in altered glycolytic metabolism, making them more radiosensitive [78] and adversely impacting the invasiveness and proliferative ability [79]. In human pancreatic cancer cells, αCHC inhibited migration and proliferation and induced cell death, performing better when applied in combination with metformin [80]. αCHC treatment of Dalton’s lymphoma cells resulted in modulation of the biophysical parameters of the tumor cell culture medium with respect to pH, nitric oxide, glucose, and lactate, inducing apoptosis and decreased cell survival [81].

## 5. Inhibitor of Pentose Phosphate Pathway

Polydatin inhibits glucose-6-phosphate dehydrogenase, the entry enzyme in the pentose phosphate pathway (PPP) causing the inhibition of NADPH production and accumulation of reactive oxygen species, inducing apoptosis. In an orthopedic metastatic model of tongue cancer, it reduced tumor size by 30% and metastasis by 80%. In a phase II clinical trial it has been proven to be safe in humans [82]. It has an anti-proliferative effect on several human tumor cell lines, such as human cervical carcinoma HeLa cells, hepatoma cell line SMMC-7721 cells, epidermal carcinoma A-431 cells and nasopharyngeal carcinoma CNE cells [83].

## 6. Inhibitor of Glutamine Metabolism

Ursolic acid is an inhibitor of ASCT2 transporters, which is a neutral transporter in the plasma membrane that preferably transports glutamine [8,84]. It is a potent anti-cancer agent for prostate cancer reducing tumor size in mouse allograft model and performed better in combination with resveratrol and curcumin [8]. It inhibited breast cancer cell proliferation by inducing G1/G2 cell cycle arrest and apoptosis through intrinsic and extrinsic apoptosis pathways [85]. In cervical cancer cell TC-1, ursolic acid activated autophagy and reduced tumor growth in vivo [86].

## 7. Inhibitor of Mitochondrial Metabolism

Gracillin, a steroidal saponin, disrupts mitochondrial complex II function by abrogating succinate dehydrogenase (SDH) activity. It induced apoptosis, suppressed ATP synthesis, and increased reactive oxygen species in several cancer cell lines derived from the lung, colorectum, prostate, pharynx, and liver with IC_50_ values of 1–5 μM. It also suppresses mutant-Kras-driven lung tumorigenesis and the growth of xenograft tumors [87].

Capsaicin and resiniferatoxin are vanilloids that induced apoptosis in human cutaneous squamous cell carcinoma (SCC) cell lines by the inhibition of mitochondrial respiration via increasing the permeability of inner mitochondrial membrane [88].

Berberine arrested cell cycle of several malignant cell lines by accumulating in mitochondria and causing mitochondrial fragmentation and depolarization and inhibiting respiration [89].

## 8. Inhibitor of Fatty Acid Synthesis

(-)-epigallocatechin-3- gallate (EGCG) the most abundant catechin compound in tea, inhibits fatty acid synthase with an IC_50_ value of 42.0 μg/mL [90]. It induced cell cycle arrest in the G1 phase and apoptosis in human colorectal cancer cells HCT-116 and SW-480 [91]. A mixture of catechins exerted a synergistic cancer cell growth inhibitory effect and antioxidant potential [92]. Catechins in combination with anticancer drugs, such as tamoxifen, COX-2 inhibitors, and retinoids showed enhanced efficacy in inducing apoptosis [93]. EGCG significantly reduced the mRNA level and activity of glycolytic enzymes and reduced breast cancer cell 4T1 growth and breast tumor weight in a dose dependent manner [94]. EGCG is also a potent inhibitor of glutamate dehydrogenase, a key enzyme in glutaminolysis process [95].

Resveratrol inhibits fatty acid synthase reversibly with IC_50_ value 6.1 μg/mL and having competitive inhibition with acetyl CoA and non-competitive inhibition with malonyl CoA [90]. It has an anti-cancer effect on several human cancers, including breast, uterine, blood, kidney, liver, eye, bladder, thyroid, esophageal, prostate, brain, lung, skin, gastric, colon, head and neck, bone, ovarian, and cervical cancer [96]. It induced apoptosis by the down-regulation of fatty acid synthase and upregulation of proapoptotic genes and suppressed growth in a xenograft model of breast cancer [97]. In some cancer cells, resveratrol exerts its cytotoxic effect by inducing intracellular palmitate accumulation that triggers lipid-mediated cell death [98]. It also acts as a radio sensitizing agent [99].

Patuletin inhibited the gene expression and activity of fatty acid synthase and induced the apoptosis of human breast cancer cell line SK-BR-3 [100].

Sea buckthorn procyanidins inhibit fatty acid synthase with a IC_50_ value of 0.087 µg/mL and reduced MDA-MB-231 cell viability with a IC_50_ value of 37.5 µg/mL [101].

Diosgenin suppresses fatty acid synthase in HER2 overexpressing breast cancer cell line, thus inducing apoptosis and enhancing paclitaxel-induced cytotoxicity [102].

[6]-Gingerol exhibits therapeutic potential by the suppression of fatty acid β-oxidation via the inhibition of fatty acid synthase and cartinitne palmitoyltranferase-1 activity in HepG2 cells [103].

Apart from these, osthole [104], amentoflavone [105], cacalol [106], cerulenin [107], gingkolic acid [108] and constituents of extra virgin olive oil (phenols, phenolic acids, flavonoids, secoirodoids) [109] have shown potential for inhibiting fatty acid synthase.

## 9. Metabolic Pathway Analysis

The mechanism of action of plant-derived compounds and their ability to modulate metabolic pathways needs to be elucidated to render them more efficacious for use as cancer therapeutics. When a compound is administered, the modulation of one component of metabolism may reprogram the whole metabolism reflected in the change of the activity of different pathways. Inhibition of glucose or glutamine transporter results in decreased uptake of the respective molecule. When glutamine transport is inhibited by ursolic acid, the cells may increase their glycolytic flux, compensating for decreased glutaminolysis, and on the other hand, inhibition of glucose uptake or glycolysis may make the cell more dependent on glutamine for energy production. Different flavonoids in conjugation with the inhibition of glucose uptake may also act as an antioxidant and have an effect on NADPH biosynthesis. Compounds such as polydatin inhibit NADPH production in the pentose phosphate pathway, and so other NADPH-producing pathways may have higher activity. Decreased pentose phosphate pathway activity may also result in decreased nucleotide biosynthesis. Agents that affect mitochondrial metabolism, such as gracillin, may induce reductive carboxylation. For this, different pathways’ activity needs to be analyzed to get an idea about the mechanism of action of a given compound.

### 9.1. Stable Isotope Tracing

Conventional metabolomics experiments are done by analyzing metabolite abundances. This has very limited implications in studying pathway activity. The abundance of a specific metabolite depends on its consumption and production. Higher abundance may indicate higher activity of the producing pathways or decreased activity of the consumption pathways. The metabolite abundance may stay the same if the rate of production complements the rate of consumption and both can have higher activity. To distinguish specific pathway activity, metabolic tracers are very useful. Stable isotope tracing or stable isotope resolved metabolomics are powerful approaches in which isotopically enriched precursors are administered as a metabolic tracer in a biological system. Introducing a metabolic tracer and analyzing the labeling pattern of downstream metabolites by mass spectrometry (MS) or nuclear magnetic resonance (NMR) spectroscopy provides information about the activity of the metabolic pathways [110]. Carefully choosing positionally labeled tracers is useful in distinguishing the activity of a specific pathway. When analyzing the activity of a specific pathway, the enrichment of the metabolic intermediates is analyzed at an isotopic steady state, where label enrichment of the metabolites is constant over time upon introduction of the tracer. Duration of the tracer incubation depends on the pathway of interest; in cultured cells, the isotopic steady state is achieved for glycolysis within just 10 min, and for TCA cycle it takes over 2 h [111]. The bioactive compounds may have multiple targets, which can make them more suitable for cancer treatment. A benefit of using stable isotope tracing metabolomics is that it can elucidate the multiple targets of a single bioactive compound. Therefore, this approach will be useful in analyzing the overall mechanism of action of a bioactive compound. The analytical methods and tools used for stable isotope tracing metabolomics have been extensively reviewed previously [110,111], and are not the focus of our review. In this review, the main focus is on how the modulation of different metabolic pathways upon administration of bioactive compounds can be analyzed using these tools. Below, we discuss the metabolic tracers used to analyze different pathways, how the results are interpreted and what the limitations are to their use. Table 2 includes information on the appropriate stable isotope tracers to employ to investigate the metabolic effects of the natural products described previously.

### 9.2. Glycolysis

Otto Warburg first observed cancer cell performing aerobic glycolysis, where even in the presence of oxygen, cancer cells limit their energy metabolism largely to glycolysis [112]. To compensate for the increased energy demand and lower energy yield of glycolysis, cancer cells increase their glucose uptake as well as glycolytic flux [113,114,115,116]. The flux of glycolysis can be measured by introducing U-^13^C glucose and measuring the percentage of incorporation of glycolysis precursors, such as fructose-1,6-bis-P, dihydroxyacetone phosphate, 3-phosphoglycerate and phosphoenolpyruvate. Compounds which inhibit glucose uptake, such as naringenin, phloretin, sylibinin, cytochalasin, curcumin, and compounds which impact different steps of glycolysis, such as mannoheptulose, sulforaphane, gossypol, konningic acid and shikonin, would directly impair glycolytic flux, which would be reflected in a lower percentage of incorporation of the glycolytic intermediates upon the introduction of U-^13^C glucose. When a specific step of glycolysis is blocked by a compound, the precursors will have higher enrichment and ^13^C incorporation will be stopped in the succeeding steps.

### 9.3. TCA Cycle

Uniformly labeled glucose (U-^13^C glucose) is usually introduced to measure the contribution of glucose to the TCA cycle intermediates, including citrate, malate, fumarate, succinate. Glucose contributes to TCA cycle via generating acetyl CoA. In the first round of the TCA cycle, M+2 intermediates will be generated. In each succeeding round, M+4 and M+6 intermediates will be generated. An alternate way of representing the oxidative TCA cycle flux from glucose is measuring M+4/M+2 ratio of citrate or other TCA cycle intermediates. Compounds that impact glucose uptake and steps of the glycolysis will limit the contribution of acetyl-CoA to the TCA cycle and thus the TCA cycle flux will be lower.

**Figure 2:**
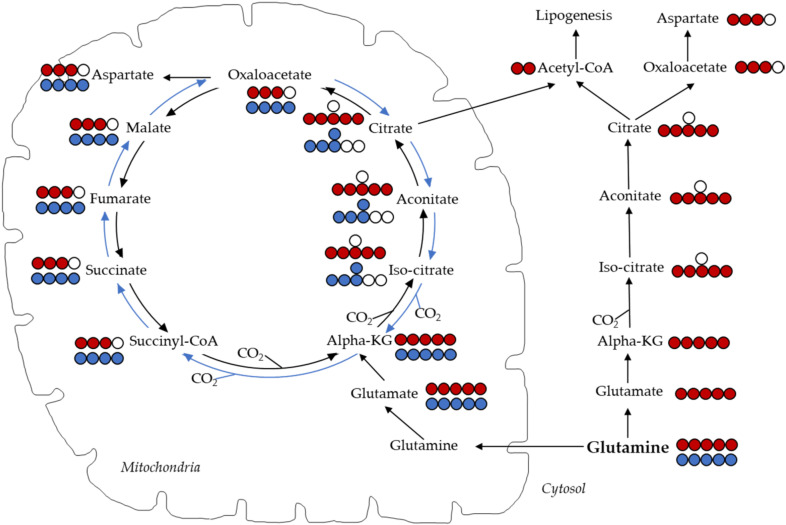
Carbon labelling TCA cycle intermediates and aspartate from U-^13^C glutamine via oxidative (carbon marked with blue) and reductive carboxylation (carbon marked with red) pathway. Oxidative glutaminolysis generates M+4-labeled TCA cycle intermediates while reductive carboxylation generates M+5 citrate followed by M+3 malate, fumarate, succinate and aspartate.

The contribution of glutamine to TCA cycle is measured by introducing U-^13^C glutamine, where glutamine is converted to alpha-ketoglutarate, which then goes through oxidative metabolism in the TCA cycle contributing 4-carbon to the TCA cycle intermediates (Figure 2). When glucose uptake or glycolysis is blocked, cells might get more dependent on glutamine and oxidative glutamine metabolism as an energy source. Glutamine can be partially oxidized in a process called glutaminolysis and add to the cellular production of NADPH and lactate. In doing so, it uses several steps of the TCA cycle and acts as a source of energy for proliferating cells. Glutamine has been proven to be a major anaplerotic precursor in proliferating glioma cells [117].

Another important pathway for metabolizing glutamine is through reductive carboxylation, where it provides carbon for lipid biosynthesis [118]. An important function of reductive carboxylation is maintaining the redox balance of mitochondria, especially during anchorage-independent growth [119]. The reductive carboxylation pathway or reverse TCA cycle can generate M+5 citrate, where U-^13^C glutamine produces alpha-ketoglutarate, which goes through the carboxylation reaction to produce M+5 isocitrate and citrate (Figure 2). This pathway can happen in both cytosol [118] or in mitochondria [120]. Generation of M+5 citrate in this pathway can later produce M+3 malate, fumarate and aspartate. A useful way of representing reductive carboxylation relative to oxidative TCA cycle activity is by measuring M+5/M+4 citrate ratio. Another way of confirming the contribution of reductive carboxylation pathway is by introducing glutamine labeled selectively at its first carbon ([1-^13^C] glutamine). If glutamine is converted to alpha-ketoglutarate and that goes through oxidative metabolism, then this carbon is lost. This carbon will only be retained in the reductive carboxylation pathway and will generate M+1 citrate and other TCA cycle intermediates. Gracillin impacts mitochondrial metabolism and thus may induce reductive carboxylation, as this is a predominant pathway in cells with impaired mitochondria for citrate synthesis [121]. Agents that block glutamine uptake, such as ursolic acid, and glutamine utilization, such as EGCG, will impair glutamine’s contribution via both oxidative and reductive carboxylation pathway.

### 9.4. Pentose Phosphate Pathway (PPP)

For increased demand of nucleotide biosynthesis and maintaining redox balance, the pentose phosphate pathway plays a vital role in proliferating cells [122]. One of the most widely used positionally labeled tracer is glucose labeled selectively at carbon 1 and 2 ([1,2-U-^13^C] glucose). Oxidative pentose phosphate pathway (oxPPP) will cause the release of carbon at position 1 of glucose as CO_2_ and subsequently generate M+1 ribose-5-P or M+1 intermediates of PPP, which can give rise to M+1 glycolytic intermediates generating M+1 lactate. If glucose goes through glycolysis that can only generate M+2 glycolytic intermediates and M+2 lactate. The flux trough PPP relative to glycolysis can be represented by the ratio of M+1 lactate/ M+1 + M+2 lactate, and this should be normalized for glucose consumption rate [123]. One limitation of this calculation is that it takes the assumption that most PPP-generated M+1 intermediates are recycled back to glycolysis, rather than being exported for nucleotide synthesis. For measuring the relative rate of oxidative to non-oxidative PPP, the ribose 5-P M+1/M+2 ratio is calculated. One limitation of this approach is that the reversibility of the PPP can generate M+2 ribose-5-P, even if it is produced via oxPPP initially. An alternative approach for measuring activity of oxPPP is the introduction of U-^13^C glucose and analyzing the sedoheptulose-7-P labeling pattern. The conjugation of labeled ribose-5-P with unlabeled xylulose-5-P can only give rise to M+5 or M+2 label enrichment in sedoheptulose-7-P in the forward direction of PPP from oxPPP generated ribose-5-P. This can estimate the ribose-5-P coming from oxPPP and hence the activity of oxPPP. Since sedoheptulose-7-P is a unique intermediate of PPP, it would provide a reliable result. The reversibility of PPP can also give rise to M+5 or M+2 sedoheptulose-7-P, but the initial direct production via oxPPP would have a major influence on percentage of incorporation.

Another way of measuring PPP is by introducing 1-^14^C glucose or 6-^14^C glucose in two separate experiments and measuring the excretion of radioactive CO_2_. Carbon number 1 of glucose is selectively released in oxPPP. Therefore, the radioactive CO_2_ released from 1-^14^C glucose relative to 6-^14^C glucose labelling experiment would indicate the flux of oxPPP. Similar results can be derived by introducing 1-^13^C glucose or 6-^13^C glucose in two separate experiments and measuring M+1 ribose-5-P. The 6-^13^C glucose would produce M+1 ribose-5-P by oxPPP, while labeled carbon number 1 of 1-^13^C glucose is lost. Comparing the enrichment of M+1 ribose-5-P from 6-^13^C glucose relative to 1-^13^C glucose would indicate oxPPP activity [124]. Polydatin blocks the initial step of oxPPP and will result in lower activity of this pathway. Agents that block glucose uptake will limit the carbon entering in the PPP and will cause reduction in its activity for the production of NADPH and nucleotides.

### 9.5. Pyruvate Carboxylase Activity

The activity of pyruvate carboxylase is strongly associated with tumorigenesis in several cancers, including breast, non-small cell lung cancer, glioblastoma, renal carcinoma, and gall bladder, and feeds the carbon skeleton of down-stream metabolites of oxaloacetate for the biosynthesis of cellular components, such as amino acid, nucleotides, and membrane lipids [125]. Heterogeneity exists, as in some cancer cells, such as MCF-7, suppression of pyruvate carboxylase activity by estrogen is associated with proliferation [126]. When U-^13^C- glucose is introduced, by the action of pyruvate dehydrogenase, labeled two carbon is incorporated in the TCA cycle via acetyl CoA and produces M+2 and M+4 TCA cycle intermediates in the first and second round of TCA cycle, respectively. Pyruvate carboxylase can incorporate one unlabeled carbon in the uniformly labeled pyruvate and generate M+3 oxaloacetate. This can go to the TCA cycle and generate M+3-labeled TCA cycle intermediates in the first round, while generating M+5 intermediates in the second round of TCA cycle. The activity of pyruvate carboxylase can be further monitored by introducing 3,4-^13^C glucose. The first carbon of pyruvate come from carbon of the 3 or 4 position of glucose. While this carbon is lost when acetyl CoA is produced from pyruvate, it can be retained if pyruvate goes through carboxylation by pyruvate carboxylase and produces M+1 oxaloacetate. This can give rise to M+1 TCA cycle intermediates, such as M+1 malate, citrate and aspartate [110]. Compounds that block glucose uptake and those which impact steps of the glycolysis pathway producing pyruvate may reduce pyruvate carboxylase activity.

### 9.6. Malic Enzyme Activity

Malic enzyme catalyzed the production of pyruvate from malate while producing NAD(P)H at the same time. There are three isoenzymes of malic enzyme—malic enzyme 1 is in the cytosol, and 2 and 3 are in the mitochondria. The malic enzyme activity can be measures by introducing U-^13^C glutamine and measuring the ratio of M+3 pyruvate/M+4 malate. Instead of pyruvate, an alternate option is to measure M+3 lactate / M+4 malate ratio. However, this gives an overall estimation of the activity of three forms of malic enzyme [124]. The activity of mitochondrial malic enzyme can be measured by isolating mitochondria and labelling with U-^13^C- glutamine or U-^13^C- malate and measuring the ratio of M+3 pyruvate or M+3 lactate/ M+4 malate. Agents that impact NADPH production via the oxPPP will induce malic enzyme for compensating the production of NADPH.

### 9.7. NAD^+^/NADH Redox State

The production of NADH can be tracked by introducing 4-^2^H-glucose, which goes through glycolysis and produce deuterium labeled NADH via the activity of glyceraldehyde-3-phosphate dehydrogenase. The ^2^H from NADH can be transferred to lactate or malate. So, the production of deuterium-labeled NADH can be monitored by measuring the M+1 enrichment in malate and lactate [127]. Koningic acid specifically block this step and will result in lower NADH level.

### 9.8. NADP^+^/NADPH Redox State

**Figure 3:**
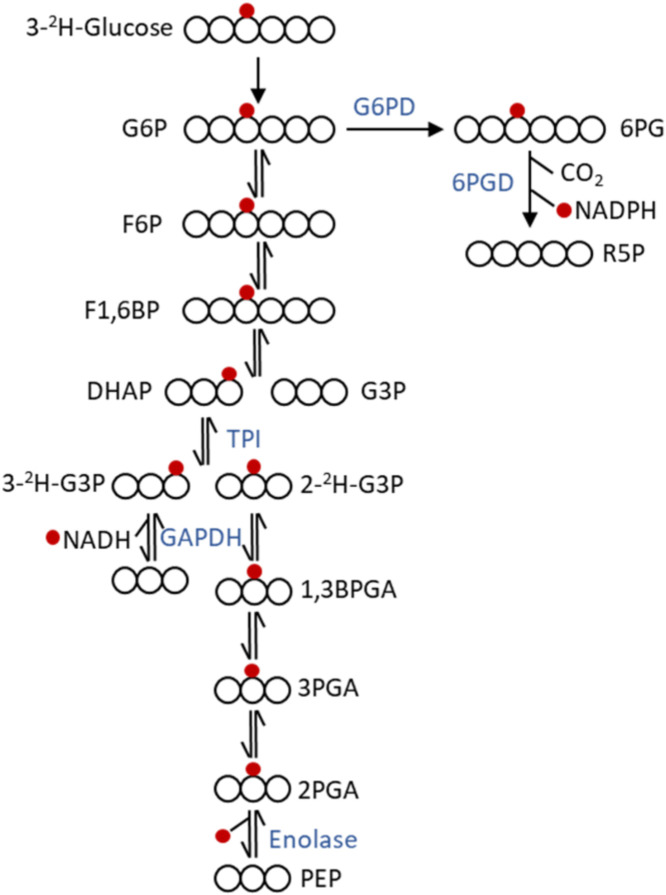
Fate of ^2^H at position 3 of glucose. 3-^2^H-glucose can produce deuterium labeled NADPH when processed through oxPPP and NADH and water when processed through glycolysis. The deuterium-labeled hydrogen is marked with a red dot. Abbreviation: G6P: glucose-6-phosphate; G6PD: glucose-6-phosphate dehydrogenase; 6PG: 6-phosphogluconate; 6PGD: 6-phosphogluconate dehydrogenase; R5P: ribose-5-phosphate; F6P: fructose-6-phosphate; F1,6BP: fructose-1,6-bis-phosphate, DHAP: dihydroxyacetone phosphate; G3P: glyceraldehyde-3-phosphate; 1,3BPGA: 1,3-bisphosphoglycerate; 3PGA: 3-phosphoglycerate; 2PGA: 2-phosphoglycerate; PEP: phosphoenolpyruvate.

The production of NADPH can be tracked by introducing 1-^2^H-glucose, which produces deuterium-labeled NADPH in the first reaction of oxPPP catalyzed by glucose-6-phosphate dehydrogenase [123]. Another way is by introducing 3-^2^H-glucose, which produces deuterium-labeled NADPH in the second reaction of oxPPP catalyzed by phosphogluconate dehydrogenase. Lewis et al. [128] showed that the production of the deuterium labeled NADPH by introducing 3-^2^H-glucose can be tracked by creating mutant IDH cells producing 2-HG and measuring the production of deuterium labeled 2-HG transferred from labeled NADPH. One limitation in using this approach is that 3-^2^H-glucose, instead of going through oxPPP, can also go through glycolysis and produce deuterium-labeled dihydroxyacetone phosphate (DHAP) intermediate (Figure 3). The hydrogen in the 3 position of DHAP is shuffled by triose phosphate isomerase enzyme and can end up in the 2 or 3 position of glyceraldehyde-3-phosphate. If 3-^2^H-DHAP produce 2-^2^H-glyceraldehyde-3-P then the deuterium labeled hydrogen is transferred in water. On the other hand, if 3-^2^H-DHAP produces 3-^2^H-glyceraldehyde-3-P then the deuterium-labeled hydrogen is transferred to NAD by the action of gyceraldehyde-3-phosphate dehydrogenase. So, the 3-^2^H-glucose can produce deuterium-labeled NADH and water through glycolysis in parallel with producing NADPH. 1-^2^H-glucose should be the preferred tracer for labelling NADPH via oxPPP. The production of NADPH from malic enzyme and isocitrate dehydrogenase can be tracked by introducing 4-^2^H-glucose, which can label malate and isocitrate and hence deuterium labeled NADPH is produced. 2,3,3-^2^H- serine can be introduced to measure deuterium labeled NADPH produced in the folate pathway. The production of deuterium-labeled NADPH can be tracked by measuring M+1 enrichment of the fatty acids. Several bioactive compounds exert their cytotoxic effect through the generation of reactive oxygen species which would present increased need for NADPH generation and different NADPH producing pathways would have higher flux for meeting the increased demand.

### 9.9. Glutathione Biosynthesis

Glutathione is produced from precursor- glutamate, glycine and cysteine. The production of glutathione can be monitored by introducing U-^13^C- glutamine (main source of glutamate) or U-^13^C- glycine. Bioactive compounds which stimulate the production of reactive oxygen species might cause increased biosynthesis of glutathione to neutralize it. Phloretin induces the biosynthesis of glutathione.

### 9.10. CoQ Biosynthesis

Coenzyme Q (CoQ) is a redox active molecule that plays a key role in the mitochondrial electron transport chain. Biosynthesis of CoQ is an important marker for mitochondrial dysfunction and oxidative stress [129]. The head group of CoQ comes from phenylalanine or tyrosine. So, the biosynthesis of CoQ can be monitored by introducing uniformly labeled phenylalanine or tyrosine. U-^13^C- glucose can also be given and label incorporation in the fatty acid chain of CoQ can be monitored, which is also an indicator of CoQ biosynthesis [130]. Compounds that impair mitochondrial oxydative phophorylation, such as gracillin, may impair CoQ biosynthesis.

### 9.11. Nucleotide Biosynthesis

**Figure 4:**
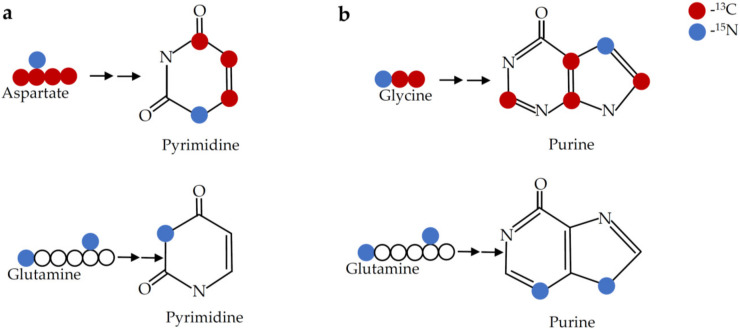
Carbon and nitrogen labelling of pyrimidine and purine rings. (**a**) When labeled with 1-^15^N, 4-^13^C- aspartate, three ^13^C and one ^15^N are incorporated into the pyrimidine ring, and when labeled with 2-^15^N- glutamine, one ^15^N is incorporated into the pyrimidine ring. (**b**) When labeled with 1-^15^N, 2-^13^C- glycine, up to four ^13^C and one ^15^N are incorporated into the purine ring, and when labeled with 2-^15^N- glutamine, two ^15^N are incorporated into the purine ring. ^13^C and ^15^N are represented with red and blue dots respectively.

Figure 4 shows the source of the carbon and nitrogen in the purine ring. Purine biosynthesis can be analyzed by introducing U-^13^C- glycine which can contribute up to four ^13^C to the purine ring, with two ^13^C derived from the direct incorporation of glycine and up to two additional ^13^C from glycine derived one carbon units.

Pyrimidine biosynthesis can be analyzed by introducing U-^13^C- aspartate and measuring M+3 uridine or UMP. Pyrimidine and purine ring biosynthesis can also be analyzed by introducing 2-^15^N- glutamine, which will contribute one and two ^15^N in the pyrimidine and purine ring, respectively. Agents that block glucose uptake or PPP will impair nucleotide biosynthesis. Agents that block TCA cycle and thus the production of aspartate will inhibit pyrimidine biosynthesis.

### 9.12. Folate Pathway

The biosynthesis of the 1C unit via the folate pathway can be analyzed via different ways. One important fact based on which biosynthesis of the 1C unit can be analyzed is that the dTTP contains 1C from cytosolic methylene THF. For analyzing the production of 1C unit in the cytosol from serine, 3-^13^C- serine is introduced and M+1 dTTP is analyzed. The third carbon of serine goes on to produce 5,10 methylene tetrahydrofolate via the action of serine hydroxymethyl transferase (SHMT). SHMT has two isoenzymes—SHMT1 is in the cytosol and SHMT2 is in the mitochondria. To distinguish the contribution of SHMT1 and SHMT2, 2,3,3-^2^H- serine is introduced. Here, M+2 dTTP is generated via the action of SHMT1 and M+1 dTTP is generated via the action of SHMT2 via a more cyclic route, where serine is cleaved in mitochondria, goes through dehydrogenation and transported to cytosol to produce 5, 10 methylene THF to get incorporated in dTTP [131]. U-^13^C- glycine can be introduced and measure M+1, M+2, M+3, M+4 ATP. Glycine can be incorporated in the purine ring generating M+2 ATP. Glycine is cleaved by the glycine cleavage system and generates 1C units in the folate pathway. The purine ring contains 1C unit from cytosolic 10-formyl-THF generating different labelling patterns in ATP. The generation of mitochondrial 1C unit can be monitored by measuring formyl-methionine M+1, as it contains 1C from mitochondrial 10-formyl-THF. Formate M+1 is also an indicator of the folate pathway activity. U-^13^C- sarcosine and U-^13^C- formate can also be introduced to measure flux through the folate pathway. When NADPH production is blocked via the oxPPP, folate pathway activity might get stimulated for compensating NADPH production.

### 9.13. Fatty Acid Biosynthesis

Fatty acid biosynthesis can happen using both glucose and glutamine as a carbon source. Using U-^13^C- glucose or U-^13^C- glutamine fatty acid biosynthesis can be monitored as they contribute to fatty acid biosynthesis via producing labeled acetyl CoA generating fatty acids labelling pattern in even numbers (M+2, 4, 6 etc). To analyze the contribution of glutamine to fatty acid biosynthesis via reductive carboxylation, 5-^13^C- glutamine is introduced. It contributes to fatty acid biosynthesis via generating M+1 acetyl CoA, resulting in labelling patterns with odd numbers in fatty acid [118]. Palmitate labelling pattern is often taken as a read out of fatty acid biosynthesis. Agents that block glucose uptake or glycolysis will impair fatty acid biosynthesis or may make cells more dependent on glutamine for fatty acid biosynthesis. Bioactive compounds such as EGCG and resveratrol directly inhibit fatty acid biosynthesis.

## 10. Conclusions

Bioactive compounds that targets different components of the metabolic process hold great potential as a safer option for the treatment of cancer. They need to be studied further in terms of how they reprogram the metabolism for their efficient use as cancer therapeutics. Carefully choosing positionally labeled stable isotope tracers could show how these compounds modulate the activity of different pathways. This will reveal their mechanism of action in cancer cells. Metabolic flux analysis in animal models and patients upon treatment with bioactive compounds could provide valuable information about the metabolic activity of bioactive compounds in a physiological state. Currently, there is lack of such studies and this could be a promising avenue of research in the future.

## Figures and Tables

**Table 1:** Effect of bioactive compounds on different metabolic processes.

Bioactive Compounds	Source	Effect	Cancers	Ref.
Naringenin (a flavonoid)	Citrus fruits, tomatoes, figs	Inhibit insulin stimulated glucose uptake	Breast, prostate, melanoma, liver	[9,10,11,12,13,14,15,16,17,18,19]
Myricetin, Fisetin, Quercetin, Isoquercitin (flavonoid)	Tomatoes, oranges, nuts, berries, tea	Inhibit GLUT2		[20]
Phloretin (dihydrochalcone)	Apple tree leaves, apple	Inhibit GLUT1 and GUT2, induce glutathione biosynthesis	Lung, liver, colon, gastric, esophageal, breast epithelial, prostate, glioblastoma	[21,22,23,24,25,26,27,28,29,30,31,32,33,34,35]
Silybinin (flavonoid)	Milk thistle	Interact with GLUT1 and GLUT4	Prostate, breast, ovary, colon, lung, bladder	[36,37,38,39,40,41,42]
Curcumin	Turmeric	Decrease GLUT1 and hexokinase II protein level	Liver, breast, pancreatic, hepatic, gastric, colorectal, prostate	[43,44,45,46,47,48,49]
Mannoheptulose	Avocado	Inhibit hexokinase		[38,50]
Sulforaphane (isothiocyanate derivative)	Cruciferous vegetables, such as broccoli	Inhibit phosphofructokinase	breast, prostate, colon, skin, lung, gastric, bladder	[51,52,53,54,55]
Koningic acid	Fungi	Inhibit glyceraldehyde-3-phohohate dehydrogenase	Neuroblastoma	[56,57,58]
Shikonin (naphthoquinone derivative)	Dried root of the plant *Lithospermum erythrorhizon*	Inhibit pyruvate kinase M2	Lung, leukemia, breast, melanoma, bladder, esophageal	[59,60,61,62,63]
Vitamin K3 and K5		Inhibit pyruvate kinase M2	Cervix	[64]
Oleanolic acid	Olive oil, garlic	Switch PKM2 to PKM1		[65]
Gossypol (polyphenolic aldehyde)	Cotton seeds	Non-selective lactate dehydrogenase inhibitor that competes with NADH	Melanoma, lung, breast, cervix, leukemia, glioblastoma, myeloma, colon, prostate	[66,67,68,69,70,71,72,73]
α-cyano-4-hydroxycinnamate (Cinnamic acid derivative)		Inhibit monocarboxylate transporter	Glioma, pancreatic, Dalton’s lymphoma	[74,75,76,77,78,79,80,81]
Polydatin	Grape, peanut, hop cones, red wines, hop pellets, cocoa-containing products	Inhibit glucose-6-phosphate dehydrogenase	Cervix, hepatoma, epidermal carcinoma, nasopharyngeal carcinoma	[82,83]
Ursolic acid	Apples, basil, cranberries, peppermint, rosemary, lavender, oregano, thyme, prunes	Inhibit ASCT2- glutamine transporter	Prostate, breast, cervix	[8,84,85,86]
Gracillin		Inhibit mitochondrial complex II	Lung, colorectum, prostate, pharynx, liver	[87]
Capsaicin	Peppers	Inhibit mitochondrial respiration	Cutaneous squamous cell carcinoma	[88]
Berberine	Root, rhizome, and stem bark of barberry, goldenseal, Oregon grape, and tree turmeric	Inhibit mitochondrial respiration		[89]
(-)-epigallocatechin-3- gallate (EGCG)	Tea	Inhibit expression of fatty acid synthase	Colorectal, breast	[90,91,92,93,94,95]
Resveratrol	Peels and seeds of grape	Inhibit fatty acid synthase	Breast, uterine, blood, kidney, liver, eye, bladder, thyroid, esophageal, prostate, brain, lung, skin, gastric, colon, head and neck, bone, ovarian, cervix	[90,96,97,98,99]
Patuletin	Pipeworts	Inhibit expression of fatty acid synthase	Breast	[100]
Sea buckthorn procyanidins	Sea buckthorn	Inhibit fatty acid synthase	Breast	[101]
Diosgenin	Fenugreek, wild yam	Inhibit fatty acid synthase	HER2 overexpressing breast	[102]
[6]-Gingerol	Ginger	Suppress fatty acid β-oxidation	Liver	[103]

**Table 2:** Use of stable isotope tracer for analyzing the metabolic activity of bioactive compounds.

Bioactive Compounds	Target	Stable Isotope Tracer
Naringenin, phloretin, sylibinin, cytochalasin, curcumin, mannoheptulose, sulforaphane, gossypol, konningic acid, shikonin	Inhibit glucose uptake and glycolysis	U-^13^C glucose
Polydatin	Inhibit PPP	[1,2-U-^13^C] glucose, 1-^13^C glucose and 6-^13^C glucose
Ursolic acid	Inhibit glutamine uptake	U-^13^C glutamine
Koningic acid	Inhibit GAPDH	4-^2^H-glucose
Phloretin	Induce glutathione biosynthesis	U-^13^C- glutamine or U-^13^C- glycine
EGCG, resveratrol, patuletin, diosgenin, [6]-gingerol	Inhibit fatty acid biosynthesis	U-^13^C glucose, U-^13^C glutamine
